# From Isolation to Genomics: Characterization of *Aspergillus uvarum* HT4 as a Novel Producer of Extracellular Tannase

**DOI:** 10.3390/jof11100722

**Published:** 2025-10-07

**Authors:** Erika Arbildi, Karen Ovsejevi, Diego Roldán, Rosario Durán, Magdalena Portela, Gabriela Garmendia, Silvana Vero

**Affiliations:** 1 Microbiology Area, Department of Biosciences, Faculty of Chemistry, Universidad de la República, Gral Flores 2124, Montevideo 11800, Uruguay; earbildi@fq.edu.uy (E.A.); droldan@fcien.edu.uy (D.R.); garmendia@fq.edu.uy (G.G.); 2 Biochemistry Area, Department of Biosciences, Faculty of Chemistry, Universidad de la República, Gral Flores 2124, Montevideo 11800, Uruguay; kovsejev@fq.edu.uy; 3 Analytical Biochemistry and Proteomics Unit, Instituto de Investigaciones Biológicas Clemente Estable & Institut Pasteur de Montevideo, Montevideo 11400, Uruguay; duran@pasteur.edu.uy (R.D.); madelon@pasteur.edu.uy (M.P.)

**Keywords:** tannase, *Aspergillus uvarum*, fungal secretome, enzyme characterization, genome sequencing, proteomics

## Abstract

Tannases (tannin acyl hydrolases, EC 3.1.1.20) are enzymes of industrial interest due to their ability to hydrolyze hydrolyzable tannins into bioactive compounds like gallic acid. In this study fungal strains capable of producing extracellular tannase were isolated and identified. From tannin-rich substrates, 24 fungal isolates were obtained, of which 17 showed tannase activity. Molecular identification based on calmodulin gene sequencing identified three species of tannase-producing black aspergilli: *Aspergillus luchuensis*, *A. niger* (formerly *A. welwitschiae*), and *A. uvarum*. The isolate *A. uvarum* HT4 exhibited the highest extracellular tannase activity (182 U/mL) and was selected for further study. Whole-genome sequencing of HT4 revealed 15 putative tannase genes, most sharing high identity with *A. uvarum* CBS 121591. Two divergent genes appeared to be acquired via horizontal gene transfer from *Aspergillus brunneoviolaceus* and *Penicillium angulare*. Proteomic analysis of the secretome confirmed the expression of two extracellular tannases. The enzyme showed optimal activity at pH 5.0–6.0 and 40–50 °C. Secretome analysis revealed hydrolytic enzymes typical of saprophytic fungi in lignocellulose-rich environments. Importantly, no biosynthetic gene clusters of major mycotoxins were detected, supporting the biosafety of HT4 for industrial applications.

## 1. Introduction

Tannases, or tannin acyl hydrolases (EC 3.1.1.20), are hydrolytic enzymes of significant industrial importance. They catalyze the cleavage of ester and depside bonds in hydrolyzable tannins, producing valuable phenolic compounds such as gallic acid, ellagic acid, and glucose [1]. Tannins, which are complex phenolic molecules of high molecular weight, are naturally present in crops like sorghum (*Sorghum bicolor*). When present in high concentrations, tannins can reduce nutrient absorption and lower the nutritional value of grains [2]. The application of tannase can transform tannin-rich grains by enhancing their digestibility, improving protein utilization, and increasing the bioavailability of essential minerals. This process broadens the potential use of these crops in agro-industrial contexts [3].

In the bioethanol industry, tannase holds particular value due to its ability to degrade tannins, especially in high-tannin grains like sorghum. Elevated tannin levels in fermentation substrates can inhibit the enzymes and microorganisms necessary for efficient ethanol production [4]. Tannase treatment reduces tannin content, thereby enhancing fermentation efficiency and potentially boosting bioethanol yield [5]. Additionally, tannase contributes to second-generation bioethanol production by breaking down phenolic inhibitors in lignocellulosic biomass, which would otherwise hinder cellulase activity [6].

Beyond its use in feed and biofuel production, tannase is widely applied in producing gallic acid and its derivatives. These compounds serve as antioxidants and antimicrobial agents in pharmaceuticals, cosmetics, and the food industry. Tannase is also employed in beverage clarification, instant tea production, and reducing bitterness in fruit juices, offering a sustainable method for processing tannin-rich products. Moreover, tannase is valuable in environmental applications, particularly in treating tannin-rich effluents from the leather tanning industry. This enzymatic approach helps to mitigate industrial pollution and reduces the environmental impact of such effluents [7].

Given its diverse applications, the pursuit of efficient tannase-producing microorganisms has become increasingly important. Fungi, especially species from the *Aspergillus* and *Penicillium* genera, are recognized as prolific producers of tannase and are frequently studied for industrial enzyme production due to their efficiency and versatility [8]. While commercial tannases are available, sourcing these enzymes from locally isolated fungi offers a sustainable and cost-effective alternative, strengthening local industries and reducing reliance on imports [9]. This study focuses on identifying and characterizing fungal strains with high tannase production potential, emphasizing local isolates to support regional biotechnological advancements. The genome of the strain with the highest tannase production capability, identified as *Aspergillus uvarum*, was sequenced, assembled, and annotated. Analysis revealed fifteen gene sequences corresponding to tannase enzymes. Furthermore, a proteomic approach was employed to examine the extracellular proteins under the specific culture conditions used in this study.

## 2. Materials and Methods

### 2.1. Chemicals

Tannic acid, gallic acid methyl ester (methyl gallate) (absolute purity 99.0%), gallic acid (97.5–102.5%), and rhodanine (≥99.0%) were purchased from Sigma Chemical Co., St. Louis, MO, USA.

An aqueous solution of tannic acid was analyzed by HPLC to quantify free gallic acid and assess the degree of hydrolysis of the reagent [10]. Upon detection of gallic acid, the tannic acid solution was purified by dialysis against distilled water to remove hydrolytic byproducts after HPLC analysis confirmed its hydrolysis. The dialysis process consisted of four water exchanges: the first two were performed 30 and 90 min after the start of the process on the first day, followed by a third exchange after 21 h, and a final exchange 7 h later. The removal of gallic acid was confirmed by HPLC, and the purified tannic acid was then stored at 4 °C until further use.

### 2.2. Isolation of Tannase-Producing Fungi

Fungi were isolated from ten samples, including compost, black tea waste, and soil rich in decomposing vegetation (e.g., leaf litter and organic residues from forested and cultivated areas) collected from various locations in Montevideo, Uruguay. To increase the number of tannase-producing fungi in the samples, enrichment cultures were performed in a liquid medium containing tannic acid as the only carbon source. Five grams of each sample was inoculated into 250 mL flasks containing 50 mL of a sterile Yeast Nitrogen Base (YNB, Difco, Franklin Lakes, NJ, USA) liquid medium supplemented with a filter-sterilized tannic acid solution to achieve a final concentration of 2%. The flasks were incubated at 28 °C for 48 h with agitation at 180 rpm. Following incubation, isolates were obtained by serial dilution and surface spread-plating on Potato Dextrose Agar (PDA; Oxoid, Basingstoke, UK), and plates were incubated at 28 °C for 72 h. Morphologically distinct fungal colonies were subcultured to obtain monosporic cultures on PDA plates. Isolates were identified to the genus level based on macro and micromorphological characteristics.

Tannase production by each fungal isolate was preliminarily confirmed using a tannic acid–agar medium prepared according to the method of Bradoo et al. [11]. Briefly, the basal medium was autoclaved at 121 °C for 15 min and supplemented with a filter-sterilized tannic acid solution to a final concentration of 1%. A 72 h old mycelial plug from each isolate was inoculated onto the medium. The plates were incubated at 28 °C for 72–96 h. The presence or absence of a hydrolysis halo around the colonies was recorded as an indicator of tannase activity.

### 2.3. Selection of Fungi with Extracellular Tannase Activity

#### 2.3.1. Extracellular Tannase Production in Liquid Medium

Fungal isolates exhibiting tannase activity on agar plates were further evaluated for extracellular tannase production in liquid culture. A 500 μL spore suspension (2.5 × 10^4^ conidia/mL) of each isolate was inoculated into 50 mL flasks containing 10 mL of YNB medium supplemented with 2% tannic acid (prepared as previously described). Cultures were incubated at 28 °C for 5 days under agitation (150 rpm). After incubation, the supernatants were collected and filtered through 0.45 μm membranes prior to tannase activity assays.

#### 2.3.2. Tannase Activity Assay

Tannase activity was measured on the filter-sterilized culture supernatants following the method described by Sharma et al. [12], with minor modifications. Methyl gallate was used as the substrate and the reaction was monitored by the increase in absorbance at λ = 520 nm, corresponding to the chromogen formation between gallic acid (released by the action of tannase on methyl gallate) and rhodanine (2-thio-4-ketothiazolidine).

Prior to the assay, the supernatants were subjected to gel filtration using PD-10 columns (Sephadex G-25, Cytiva, Uppsala, Sweden) to remove the gallic acid accumulated during fungal growth. The resulting gel-filtered supernatant was defined as the “tannase extract” (TE). Before activity determination, the TEs, methyl gallate solution, and citrate buffer were pre-incubated at 30 °C for 15 min. The enzymatic reaction was initiated by mixing 50 μL of TE with 50 μL of 0.01 M methyl gallate in 0.05 M citrate buffer pH 5.0, followed by incubation at 30 °C for 30 min. After incubation, 60 μL of methanolic rhodanine (0.667%) was added, and the mixture was incubated for an additional 5 min at 30 °C. Since the rhodanine–gallic acid complex forms only in basic conditions, after the enzymatic degradation of methyl gallate, 40 μL of 0.5 M KOH was added, and the tubes were incubated for another 5 min. The reaction mixture was diluted with 800 μL of distilled water, incubated for 10 min at 30 °C, and finally the absorbance was measured at λ = 520 nm. A negative control, consisting of the assay mixture without the substrate, was included for background correction.

One enzyme unit of tannase activity was defined as the amount of enzyme required to release 1 μmol of gallic acid per minute under standard reaction conditions.

All enzymatic extracts were performed in duplicate, and the results were analyzed using the Kruskal–Wallis test, followed by post hoc comparisons of means using Infostat [13].

Based on the extracellular tannase activity levels, the TE that showed the highest activity and the strain that produced it were chosen for further studies.

### 2.4. Molecular Identification of Tannase Producers

#### 2.4.1. DNA Extraction

Isolates were inoculated in PDA and incubated at 28 °C for 3 days. Total DNA was extracted using Quick-DNA Fungal/Bacterial Miniprep kit (Zymo Research, Irvine, CA, USA). The quality and quantity of DNA were estimated by agarose gel electrophoresis [14].

#### 2.4.2. Calmodulin Gene Amplification

Amplification of the calmodulin gene was carried out in 25 μL of reaction mixture containing 19.6 μL milli-Q water, 2.5 μL Buffer 10X (Fermentas International Inc., Burlington, ON, Canada), 0.5 μL of each primer 25 μM, 0.8 μL of dNTPs 10 mM (Fermentas International Inc., Canada), 0.1 μL of Taq polymerase 5 U/μL (Fermentas International Inc., Canada), and 1 μL of DNA (50 μg/mL). The primers used in the reaction were CF1 (5′-GCCGACTCTTTGACYGARGAR-3′) and CF4 (5′-TTTYTGCATCATRAGYTGGAC-3′) according to Garmendia, G & Vero, S [15]. PCR was performed in a MultiGene Mini thermocycler (Labnet International, Inc., Edison, NJ, USA) under the following conditions: initial denaturation at 94 °C for 2 min, followed by 35 cycles of denaturation at 96 °C for 30 s, annealing at 51 °C for 60 s, extension at 72 °C for 60 s, and a final extension at 72 °C for 5 min. PCR products were separated on 0.8% agarose gels (Agarose I™, Amresco, Solon, OH, USA) containing gel red (Biotium, Fremont, CA, USA) and DNA bands were visualized under UV light.

#### 2.4.3. Phylogenetic Analysis of Calmodulin Sequences

Nucleotide sequences of the PCR products were obtained at Macrogen (Macrogen Inc., Seoul, Republic of Korea). The sequences were aligned using MEGA version 7, manually adjusted, and compared against sequences from type strains from the NCBI database via BLAST (Basic Local Alignment Search Tool at NCBI (https://blast.ncbi.nlm.nih.gov, accessed on 10 April 2025)). Phylogenetic analysis of the calmodulin sequences from the selected isolates was performed with MEGA version 7. Evolutionary distances were estimated using the maximum likelihood method and the Kimura two-parameter model. The percentage of trees in which the associated taxa are grouped together is displayed next to the branches. Initial trees for heuristic searches were automatically generated by applying the neighbor-join and BioNJ algorithms to a pairwise distance matrix calculated with the maximum composite likelihood (MCL) approach, followed by selecting the topology with the highest log-likelihood value. Clade stability was assessed through 1000 bootstrap replicates.

### 2.5. Whole-Genome Sequencing, Assembly, and Annotation of the Selected Isolate

Genomic DNA of HT4 was extracted from fungal mycelia grown for 3 days on PDA plates using the Quick-DNA Fungal/Bacterial Miniprep Kit (Zymo Research). The DNA concentration was quantified using a Qubit™ fluorometer with the Qubit™ dsDNA HS Assay Kit (Invitrogen, Waltham, MA, USA) and normalized to 0.2 ng/µL. The normalized DNA was then submitted to Novogene for sequencing on the Illumina NovaSeq X Plus platform (PE150 configuration, San Diego, CA, USA).

Raw sequencing reads were pre-processed to remove adapter sequences and trim low-quality bases using Trimmomatic [16]. The cleaned paired-end reads were assembled de novo using SPAdes v3.15.3 [17] with default parameters. To assess completeness of the assembled genome, BUSCO v5 [18] was employed and gene prediction was performed with the AUGUSTUS tool (v3.5.0) [19]. Predicted coding sequences (CDSs) were functionally annotated using InterProScan v5.25–64.0 [20] with the Pfam database [21]. The assembled genome was analyzed using the antiSMASH pipeline version 6.1.1 implemented in Galaxy (Galaxy Version 6.1.1+galaxy1) to identify biosynthetic gene clusters (BGCs) involved in the production of secondary metabolites.

#### Identification and Phylogenetic Analysis of Tannase Sequences

Putative tannase sequences were further analyzed using BLAST (Basic Local Alignment Search Tool) at NCBI https://blast.ncbi.nlm.nih.gov (accessed on 10 May 2025) against the NCBI non-redundant (nr) database and UniProt KB to retrieve the most similar sequences. Maximum likelihood (ML) phylogenetic trees were constructed using those sequences that did not show similarity to *Aspergillus uvarum* tannases in order to compare them with their closest homologs retrieved from the databases. Phylogenetic analyses were performed in MEGA v11 using the WAG gamma substitution model and 1000 bootstrap replicates to assess branch support. To assess potential function, these amino acid sequences (not similar to *A. uvarum* sequences) were submitted to InterProScan v5 to look for conserved domains corresponding to the tannase/feruloyl esterase family. The presence of the catalytic serine hydrolase motif GXSXG was evaluated manually.

### 2.6. Characterization of TE Produced by the Selected Isolate

#### 2.6.1. Protein Determination

Protein content was determined using the BCA assay [22]. Bovine serum albumin was used as the standard.

#### 2.6.2. Effect of pH and Temperature on Tannase Activity

The effect of pH and temperature on enzyme activity was evaluated using the selected TE. Tannase activity was assessed at 30 °C within the range of pH 4.0–7.0 and at pH 5.0, changing the reaction temperature from 4 to 60 °C.

#### 2.6.3. Verification of Tannase Activity by Zymogram

Tannase activity was further verified using native gel electrophoresis. TEs with the highest activity produced by *A. uvarum* strains HT3 and HT4 were concentrated in Vivaspin 500 concentrators (Vivaspin 500, 50kDa MWCO, GE HealthCare, Chicago, IL, USA) and then loaded onto polyacrylamide gel with a gradient of acrylamide concentrations (4–20%). A native PAGE electrophoresis was run in a Mini-PROTEAN Tetra Vertical Electrophoresis Cell (Bio-Rad, Hercules, CA, USA) under cool conditions at pH 8.3. After the electrophoresis, the gel was removed and washed for 30 min with 100 mL of 0.05 M citrate buffer pH 5.0, followed by a 10 min wash with the same buffer, with constant shaking at 70 rpm. Then, the gel was incubated with 0.5% (*w*/*v*) tannic acid in 50 mL 0.05 M citrate buffer pH 5.0 for 30 min at 28 °C. Tannic acid solution was discarded and, subsequently, the gel was rinsed with the same buffer, replaced with methanolic rhodanine (0.667%), and incubated at 28 °C to visualize the tannase activity on the gel.

#### 2.6.4. Protein Identification by Nano LC-MS/MS

The HT4 TE was concentrated in a vacuum concentrator (Thermo Scientific™ Savant™ SpeedVac™ SPD1010, Waltham, MA, USA) and then loaded onto polyacrylamide gel with a gradient of acrylamide concentrations (4–20%). A native PAGE electrophoresis was run in a Mini-PROTEAN Tetra Vertical Electrophoresis Cell (Bio-Rad) under cool conditions at pH 8.3. Following electrophoresis, the gel was removed and fixed for 30 min in a solution containing 10% acetic acid, 40% ethanol, and 50% distilled water. The fixing solution was removed and the gel was stained with Coomassie Brilliant Blue G-250 for 72 h, according to the protocol of colloidal staining described by Neurhoff et al. [23,24]. The protein bands corresponding to tannase enzymes were identified based on their migration pattern in the previously performed zymogram. These bands were subsequently excised from the Coomassie-stained gel and processed for identification by mass spectrometry (MS).

Additionally, a second electrophoresis was performed under the same conditions, but the run was intentionally stopped before completion. In this case, instead of excising well-defined bands, a continuous portion of the gel containing a diffuse protein smear was excised and processed for mass spectrometry analysis in order to identify the proteins present in the enzymatic extract (TE). This procedure was carried out in quadruplicate.

Sample processing for MS analysis was performed as previously described [25]. Briefly, the bands were reduced with 10 mM DTT at 56 °C for 1 h, followed by cysteine alkylation with 50 mM iodoacetamide at room temperature for 45 min. In the gel, tryptic digestion was performed overnight using sequencing-grade trypsin (Promega, Madison, WI, USA). Peptides were extracted with 60% acetonitrile/0.1% trifluoroacetic acid, concentrated by vacuum drying, and resuspended in 0.1% formic acid.

Peptides were analyzed on a nano-HPLC (UltiMate 3000, Thermo, Waltham, MA, USA) coupled to a hybrid quadrupole–orbitrap mass spectrometer (Orbitrap Exploris 240, Thermo). For analysis of supernatant proteins, an elution gradient from 4% to 35% B over 150 min was used (A: 0.1% formic acid; B: 0.1% formic acid in acetonitrile) at a constant flow rate of 200 nL/min. For specific bands, a shorter gradient (from 4 to 35%B in 45 min) was used. MS data acquisition was performed in positive ion mode and in a data-dependent manner, with full MS scans followed by HCD fragmentation of the 20 most intense ions in each segment. Peptide spectrum matching and protein identification were performed using PatternLab for Proteomics V [26], with a target-reversed database generated from the whole-genome sequencing data of the strain. Met oxidation was included as a variable modification and Cys alkylation as fixed. Results were filtered to achieve <1% FDR at the protein level.

The mass spectrometry proteomics data have been deposited to the ProteomeXchange Consortium via the PRIDE 1 partner repository with the dataset identifier PXD066288.

#### 2.6.5. Secretory Proteins in the HT4 Enzymatic Extract from HT4

Secretory proteins were predicted using SignalP 5.0 [27]. The normalized spectral abundance factor (NSAF) for each identified protein in the quadruplicate proteomic analysis HT4 secretome was calculated, and the values were summed by functional class. The contribution of each class was then expressed as a percentage of the total NSAF.

## 3. Results

### 3.1. Isolation and Screening of Tannase-Producing Fungi

A total of 24 fungal isolates were obtained from enrichment cultures grown in the presence of tannic acid, belonging to the genera *Aspergillus*, *Penicillium*, *Rhizopus*, and *Paecilomyces*. These isolates were screened for tannase production using a plate assay method. Seventeen of the isolates, representing 70.8%, exhibited detectable tannase activity when cultured with 1% tannic acid as the sole carbon source.

### 3.2. Extracellular Tannase Production in Liquid Medium

**Figure 1:**
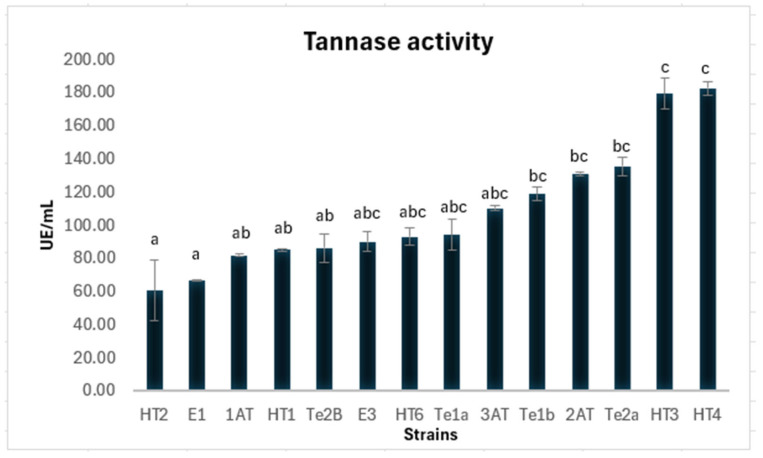
Tannase activity of different strains. Enzyme activity was determined using 50 mM citrate buffer (pH 5.0) and 1 mM methyl gallate as substrate under standard assay conditions. Results are presented as mean ± standard deviation. Different letters indicate statistically significant differences according to Kruskal–Wallis test followed by a multiple comparisons test (*p* < 0.05).

Extracellular tannase production was evaluated for the 17 fungal isolates that exhibited tannase activity in the plate assay. Of these, 14 isolates demonstrated extracellular tannase activity in a culture medium containing tannic acid as the sole carbon and energy source. The results, presented in Figure 1, show the average enzymatic activity of the TEs along with their respective standard deviations. Among the isolates, strain HT4 displayed the highest enzymatic activity (182 U/mL) and was therefore selected for detailed characterization. HT3 also demonstrated promising activity and should be examined in subsequent studies.

### 3.3. Molecular Identification of Tannase Producers

**Figure 2:**
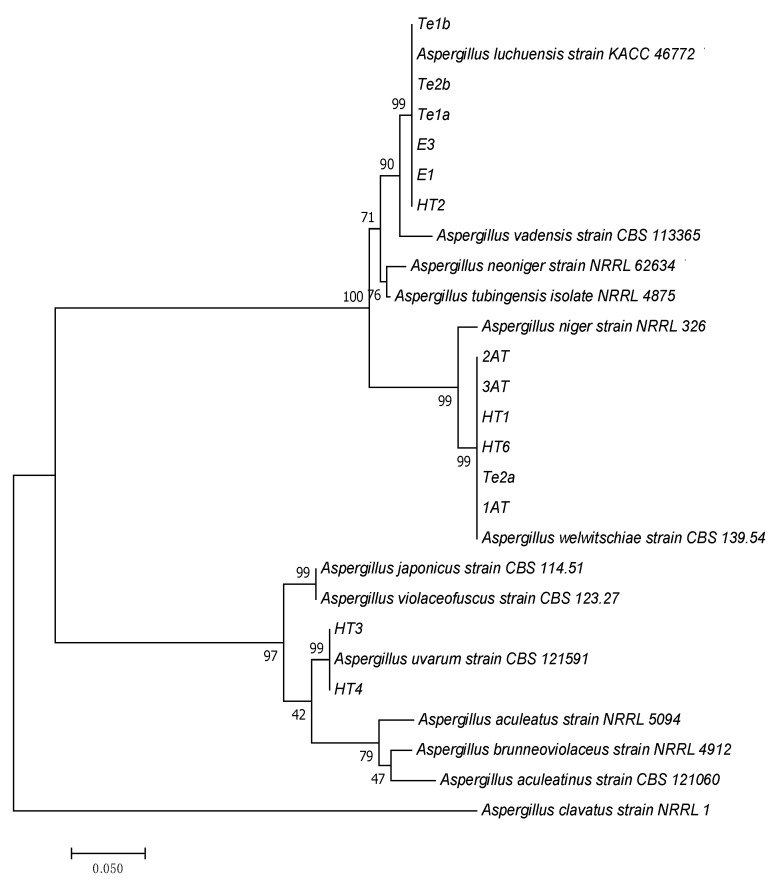
Phylogenetic tree based on calmodulin gene sequences of tannase-producing Aspergillus isolates. The tree was constructed using the maximum likelihood method with the Kimura 2-parameter model in MEGA 7. Homologous sequences from type strains were retrieved from GenBank. Bootstrap values (1000 replicates) are shown at the branch nodes.

All the strains exhibiting extracellular tannase activity belonged to the black Aspergilli group. They were molecularly identified by analyzing DNA sequences encoding the calmodulin gene. These sequences were compared with homologous sequences from type strains available in the GenBank database. A phylogenetic tree was constructed using the query sequences and their closest matches retrieved from the database (Figure 2).

Based on the phylogenetic analysis, the strains were classified into three distinct species: six strains were identified as *Aspergillus luchuensis*, six as *Aspergillus welwitschiae*, and two as *Aspergillus uvarum*. However, according to the recent revision by Visagie et al. [28], *A. welwitschiae* must be considered a synonym of *A. niger*.

### 3.4. Genome Analysis of the Selected Strain

The final draft assembly of HT4 comprised 231 contigs, with a total genome size of 36,147,491 bp, an N50 contig size of 541,142 bp (maximum contig length: 1,971,308 bp), and a G+C content of 50.8%. The assembly was generated from 4 Gbp of Illumina sequencing data (27,973,698 reads), providing 191× coverage. Genome completeness was estimated at 98.8%, comprising 98.4% single-copy genes, 0.4% duplicated genes, 0.5% fragmented genes, and 0.7% missing genes. A total of 10,796 protein-coding sequences (CDSs) were predicted, among which 15 were identified as tannase-coding genes. This Whole Genome Shotgun project has been deposited in DDBJ/ENA/GenBank under the accession JBLHEK0000000001.

#### 3.4.1. Tannase Sequences in HT4 Genome

**Figure 3:**
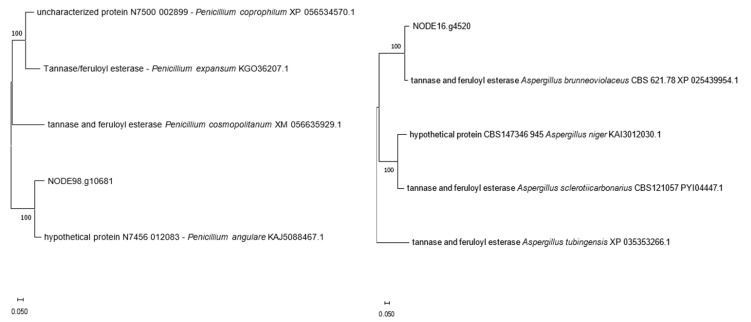
Phylogenetic tree of non-*Aspergillus* tannase sequences, constructed by MCL method.

Comparative analysis using protein databases revealed that 13 of the 15 tannase sequences identified in the HT4 genome shared between 95% and 100% identity with tannases from *Aspergillus uvarum* CBS 121591. The remaining two sequences exhibited the highest similarity to tannases from *Aspergillus brunneoviolaceus* CBS 621.78 (92.31% identity) and a hypothetical protein from *Penicillium angulare* IBT 30069 (88.20% identity) (Table 1). A phylogenetic analysis was performed to investigate the evolutionary relationships of these two divergent sequences, with their closest homologs retrieved from public databases (Figure 3). Unrooted phylogenetic trees were constructed in MEGA 11 using the nearest homologs (high sequence identity and coverage) to assess the immediate phylogenetic proximity of both tannases. In this framework, the two proteins appear displaced from the expected host-species clade, a pattern consistent with horizontal acquisition; no specific donor or transfer direction is inferred.

Although BLASTp analysis indicated that one of these sequences aligned most closely with a hypothetical protein from *P. angulare*, further functional characterization strongly supported its annotation as a tannase. InterProScan detected the presence of the tannase/feruloyl esterase domain (IPR011118) and the Pfam tannase family domain (PF07519). Additionally, the sequence was classified within the feruloyl esterase B-related subfamily (PTHR33938) according to the Panther database. Manual inspection revealed a conserved GXSXG motif (GCSGG), which is characteristic of the catalytic site in serine hydrolases.

#### 3.4.2. Identification of Secondary Metabolite Gene Clusters

Genome mining using antiSMASH (Galaxy Version 6.1.1+galaxy1) identified a total of 60 putative biosynthetic gene clusters (BGCs) potentially involved in secondary metabolite biosynthesis. These included clusters encoding non-ribosomal peptide synthetases (NRPS, 23 clusters), type I polyketide synthases (T1PKS, 18), terpenes (7), hybrid NRPS–PKS systems (4), and indole alkaloids (3), as well as siderophores, β-lactones, and other biosynthetic types. Notably, six clusters exhibited 100% identity with experimentally characterized BGCs, indicating a strong biosynthetic capacity of this strain (Table 2). A comprehensive list of all predicted clusters, including cluster types, genomic coordinates, and predicted products, is available in Supplementary Table S1.

Importantly, no complete biosynthetic gene clusters associated with the production of major fungal mycotoxins—such as aflatoxins, ochratoxins, citrinin, or fumonisins—were detected. However, region 21.4 was predicted to encode an indole-derived NRPS/NRPS-like BGC, showing high similarity to clusters known to produce diketopiperazines, including histidyl-tryptophanyl-diketopiperazine, dehydrohistidyl-tryptophanyl-diketopiperazine, roquefortine D, roquefortine C, glandicoline A/B, and meleagrine. Among these, roquefortine C is a well-known secondary metabolite with moderate neurotoxic potential, primarily reported in *Penicillium* species.

In addition, two other regions—18.1 and 91.1—exhibited partial similarity (66% and 57%, respectively) to gene clusters involved in the biosynthesis of patulin and cyclopiazonic acid (CPA). In region 18.1, only the T1PKS backbone was present, while key tailoring genes (e.g., *patK*, *patN*) were absent. Similarly, region 91.1 contained an NRPS module but lacked essential biosynthetic genes (*cpzA–cpzD*), suggesting that both clusters are incomplete, likely non-functional, and may represent evolutionary remnants.

Principio del formulario

Final del formulario

The table includes the type of BGC, predicted metabolite, reported bioactivities based on the literature, and potential biotechnological or pharmaceutical applications. Superscript numbers refer to supporting references.

### 3.5. Characterization of TE Produced by the Selected Isolate

#### 3.5.1. Protein Determination

The protein concentration of the HT4 TE, determined by the BCA assay, was 0.0149 ± 0.0002 mg/mL.

#### 3.5.2. Effect of pH and Temperature on Enzyme Activity

**Figure 4:**
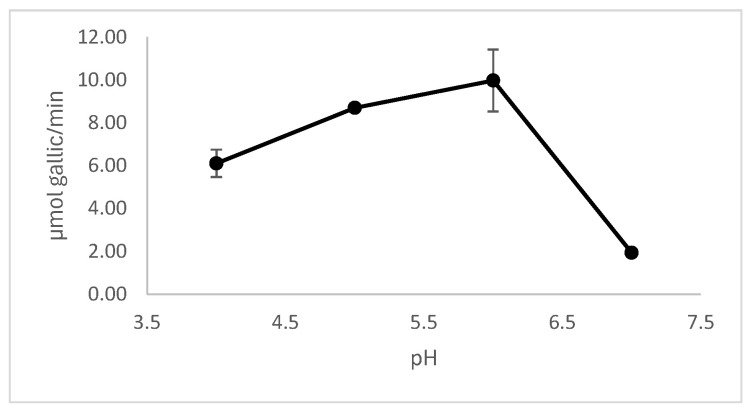
Effect of pH on enzymatic activity. The enzyme activity was measured at various pHs under standard assay conditions. Results are expressed as mean ± standard deviation.

The effect of pH on the enzymatic activity of the selected tannase extract was evaluated. The optimum pH range was 5.0–6.0, with no significant differences in activity between these pHs (Figure 4).

**Figure 5:**
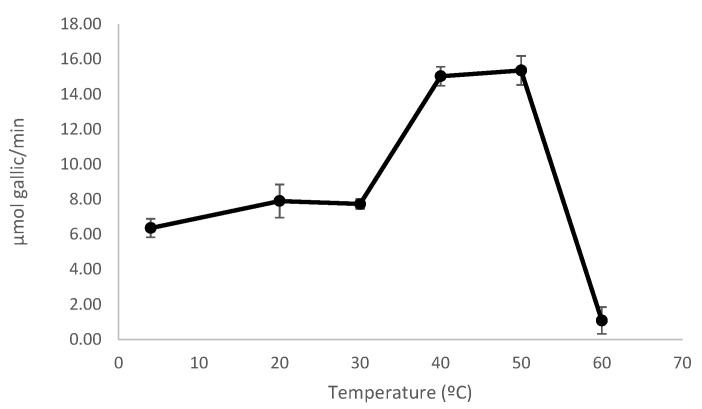
Effect of temperature on the enzymatic activity. The enzyme activity was measured at various temperatures under standard assay conditions. Results are expressed as mean ± standard deviation.

The optimal temperature for enzymatic activity was found to be between 40 °C and 50 °C (Figure 5). At these temperatures, a two-fold increase in tannase activity was observed compared to that at 30 °C. As mentioned in Section 2.3.2, tannase activity was measured following the method previously reported by Sharma et al. [12], where tannases from *Aspergillus niger* and *Aspergillus oryzae* were used to optimize the assay. In the present study, tannase was produced by a strain from a different species and exhibited distinct biochemical characteristics. Therefore, in future studies, the activity assay will be evaluated at temperatures between 40 °C and 50 °C.

#### 3.5.3. Tannase Verification by Zymogram

**Figure 6:**
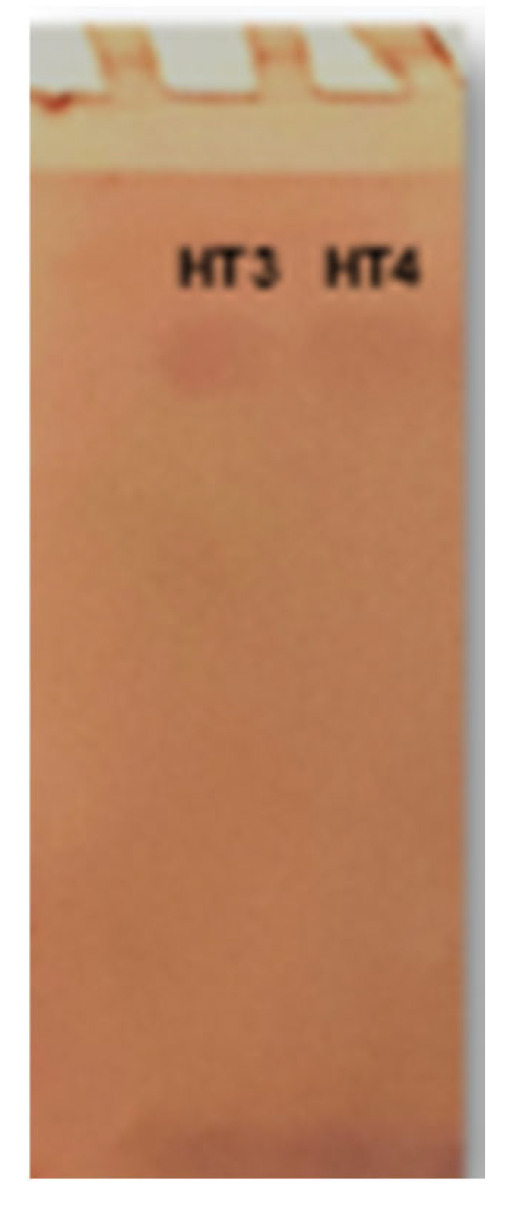
Zymographic analysis showing tannase activity bands in lanes corresponding to *A. uvarum* strains HT3 and HT4 respectively.

Concentrated tannase extract from HT3 and HT4 (*Aspergillus uvarum* strains) were assessed for enzyme activity using the zymogram procedure described in the Section 2. Tannase activity was identified as a single band in the concentrated cell-free extracts. The zymogram assay confirms that both strain extracts contain an enzyme capable of hydrolyzing the ester bonds of tannic acid (Figure 6).

### 3.6. Protein Identification by Nano LC-MS/MS

Analysis of the selected band corresponding to tannase led to the statistically significant identification of two tannases (NODE_18_length_653725_cov_92.2. g4952 and NODE_60_length_181120_cov_91.6. g9637) identified with 93 and 41 spectra, respectively (Table 3). From the protein identification, the two peptides were assigned to tannases from *Aspergillus uvarum* CBS 121591.

**Figure 7:**
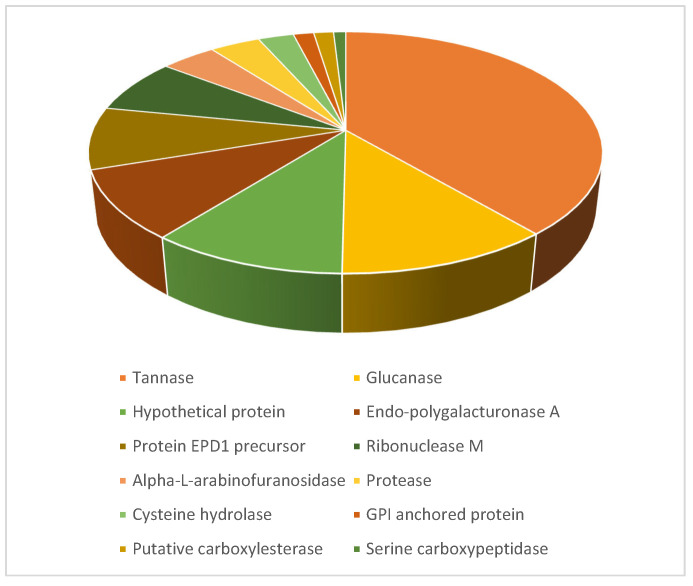
Relative abundance of proteins in the *Aspergillus uvarum* secretome across different protein classes, expressed as NSAF (normalized spectral abundance factor) values. Protein categories were defined based on annotations obtained through BlastP searches against the NCBI database.

In addition, the analysis of the entire supernatant confirmed the presence of these two enzymes. A total of 35 proteins were identified (Supplementary Materials), 16 of which were classified as secretory proteins and assigned to different functional categories (Figure 7). All identified proteins showed high sequence similarity to homologs encoded in the genome of *A. uvarum* CBS121591.

## 4. Discussion

The enrichment strategy using tannic acid as the sole carbon source proved effective for the selective isolation of tannase-producing fungi. A total of 24 isolates were obtained, with a high proportion (70.8%) exhibiting tannase activity in agar plate assays. This result highlights the metabolic versatility of fungal genera such as *Aspergillus*, *Penicillium*, *Rhizopus*, and *Paecilomyces*, which are known to include strains capable of producing hydrolytic enzymes involved in polyphenol degradation [35].

Further screening for extracellular enzyme production in the liquid culture revealed that 14 of the 17 plate-positive isolates secreted measurable levels of tannase into the medium. These results confirm that most tannase-producing fungi isolated can secrete the enzyme efficiently into the extracellular environment, a critical feature for potential industrial applications. Notably, all the strains exhibiting extracellular tannase activity belonged to the black Aspergilli group, in agreement with previous studies that have identified *Aspergillus* species as prominent producers of tannase [36,37,38].

The highest tannase activity was observed in the strain HT4 identified as *Aspergillus uvarum*, a species that has been relatively underexplored for tannase production. This strain exhibited an enzymatic activity of 182 (±4) UE/mL, similar to the activity reported by Lal, D., & Gardner, J. [39] for a selected strain of *Aspergillus niger* in the same reaction conditions.

Biochemical characterization of the enzyme extract showed an optimal pH range between 5.0 and 6.0 and a temperature optimum between 40 and 50 °C, which aligns with previously reported fungal tannases [40,41,42,43]. Zymogram analysis revealed that the tannase activity was localized in a specific region of the gel, which was observed after the sample underwent polyacrylamide gel electrophoresis. This finding highlights the presence of active tannase in the analyzed sample and provides insight into the enzyme’s electrophoretic behavior.

*Aspergillus uvarum* appears as a safe alternative tannase producer as it has not been reported to produce major mycotoxins such as ochratoxins and fumonisins vinculated to other species within the *Aspergillus* section Nigri [44]. Genomic analysis using antiSMASH confirmed that strain HT4 lacks the biosynthetic gene clusters responsible for these major mycotoxins, reinforcing its potential as a safe and efficient tannase producer. Although partial similarity was found with patulin and cyclopiazonic acid clusters, the absence of essential core biosynthetic genes indicates that these regions are likely non-functional or degenerate. Interestingly, one biosynthetic gene cluster (region 21.4) displayed 100% similarity to pathways involved in the biosynthesis of indole-derived diketopiperazines, including compounds such as roquefortine D, meleagrine, and glandicolines. Among these, *roquefortine C* is a well-characterized secondary metabolite with moderate neurotoxic activity. It is primarily produced by *Penicillium* species and is considerably less toxic than major foodborne mycotoxins [33]. Although no prior reports exist of *roquefortine C* production in *A. uvarum*, the presence of this cluster in HT4 suggests a latent biosynthetic capacity that should be further investigated. Its actual expression under tannase-inducing conditions remains unknown and would need experimental validation. Nonetheless, the relatively low toxicological concern associated with roquefortine-type metabolites, combined with the absence of high-risk clusters, supports the continued investigation of *A. uvarum* HT4 for safe industrial use.

In addition to confirming the absence of harmful metabolites, antiSMASH revealed that the genome of *A. uvarum* HT4 harbors 60 predicted biosynthetic clusters, reflecting substantial biosynthetic potential. Among them, five clusters showed 100% similarity to well-characterized metabolites, including clavaric acid (a farnesyltransferase inhibitor with anticancer potential) [29], okaramine D (an insecticidal compound targeting glutamate-gated chloride channels) [30], cyclic depsipeptides (with antimicrobial and anticancer activity) [31], naphtho-γ-pyrones (antimicrobials) [32], and monascorubrin (a natural pigment with antioxidant activity) [34]. These findings highlight the added value of HT4 not only as a tannase producer but also as a potential source of pharmacologically and industrially relevant secondary metabolites. Moreover, a significant portion of the predicted BGCs (55 out of 60) showed no similarity to any known clusters, suggesting the existence of novel or uncharacterized metabolic pathways. These orphan clusters may encode entirely new secondary metabolites with unexplored bioactivities, offering exciting prospects for future bioprospecting and functional genomics studies.

Final del formulario

The genome analysis of the selected strain HT4 provides valuable insights into its genetic architecture and the molecular basis of its tannase production capabilities. The final draft assembly, comprising 231 contigs with a total genome size of 36.1 Mbp, represents a high-quality genome with 98.8% completeness, indicating that the majority of the genome has been successfully captured. The high mapped coverage of 191x ensures the reliability of the assembly, while the N50 contig size of 541.1 kbp and the maximum contig length of 1.97 Mbp reflect a well-assembled genome with large, continuous sequences. The identification of 10,796 protein-coding sequences in the HT4 genome highlights its genetic complexity and metabolic potential. Among these, 15 putative tannase-coding genes were detected. These multiple coding sequences likely enable the production of a variety of tannase isoforms, each potentially specialized for different substrates or environmental conditions, enhancing the fungus’s ability to degrade a wide range of tannin compounds.

This phenomenon of multiple genes encoding enzymes with the same activity has been observed in other fungi. For instance, Busk et al. [45] reported that some cellulose-degrading fungi, including *Aspergillus* spp., on average, possess 11 genes coding for β-glucosidases. They proposed that this genetic diversity allows the fungi to produce enzymes with distinct properties, making them suitable for the degradation of cellulose in various plant cell wall materials and under different environmental conditions. Similarly, the presence of multiple tannase genes in *A. uvarum* may reflect an evolutionary adaptation to efficiently hydrolyze diverse tannin structures in its ecological niche.

The majority of these genes (13 out of 15) showed 100% high homology to tannase sequences from *Aspergillus uvarum* CBS 121591, suggesting that they were inherited vertically or arose through gene duplication events within the *A. uvarum* lineage. However, the presence of two tannase genes with homology to sequences from *Aspergillus brunneoviolaceus* CBS 621.78 (92.3%) and *Penicillium angulare* IBT 30069 (87.2%) indicates possible horizontal gene transfer (HGT) events. These HGT events likely provided HT4 with additional tannase genes, enhancing its ability to degrade a broader range of tannins and adapt to specific environmental conditions. The acquisition of these genes from distantly related species highlights the evolutionary flexibility of fungi and their ability to integrate foreign genetic material to improve their metabolic capabilities.

Despite the presence of 15 tannase genes in the genome, only two tannases were detected in the culture supernatants under the experimental conditions used in this study, as confirmed by LC–MS/MS analysis. This discrepancy between gene presence and protein expression suggests that not all tannase genes are actively transcribed and translated under the given conditions. The detection of only two tannases may be due to regulatory mechanisms that control gene expression in response to specific environmental cues, such as the type and concentration of tannins present in the medium. Alternatively, the other tannase genes may be expressed at low levels, under different conditions, or in response to other stressors, highlighting the need for further studies to explore the regulatory networks governing tannase expression in HT4.

The characterization of the HT4 secretome under tannin-rich conditions provided valuable insight into the metabolic strategies employed by this strain. Proteomic analysis identified 35 proteins in the culture supernatant, 16 of which were predicted to be secretory based on SignalP analysis. The functional classification of these secretory proteins revealed a predominance of hydrolytic enzymes, such as esterases and glycoside hydrolases, including the two confirmed tannase isoforms. This enzymatic profile reflects fungal adaptation to metabolize complex phenolic substrates, suggesting that *A. uvarum* HT4 remodels its extracellular environment to access carbon sources bound within recalcitrant plant polymers. The observed protein composition is characteristic of saprophytic fungi occupying lignocellulose-rich ecological niches [46]. Moreover, the selective expression of only a subset of the predicted tannases supports the hypothesis of environmentally responsive regulation and highlights the dynamic nature of the HT4 secretome.

In conclusion, the combined phenotypic, enzymatic, and genomic data presented here underscore the potential of *Aspergillus uvarum* HT4 as a robust and safe producer of tannase. Its lack of major mycotoxin biosynthetic pathways, together with a diverse secondary metabolite repertoire and multiple tannase gene candidates, makes it a promising candidate for biotechnological development. Future work should aim to optimize culture conditions, explore expression of cryptic BGCs, and evaluate the full metabolic potential of HT4 across industrial, food, and pharmaceutical applications.

## Figures and Tables

**Table 1:** Putative tannase sequences identified in the genome of *Aspergillus uvarum* HT4 and their closest homologs.

Identifier	Length (AA)	Identity (%)	Homolog Source
NODE_1.g376	512	95.20	*Aspergillus uvarum* CBS 121591 XP_025490290.1
NODE_3.g1172	537	100	*Aspergillus uvarum* CBS 121591 XP_025488689.1
NODE_9.g2866	543	97.18	*Aspergillus uvarum* CBS 121591 XP_025492021.1
NODE_11.g3384	1477	99.60	*Aspergillus uvarum* CBS 121591 XP_025487606.1
NODE_11.g3515	548	100	*Aspergillus uvarum* CBS 121591 XP_025485684.1
NODE_16.g4469	588	99.83	*Aspergillus uvarum* CBS 121591 XP_025491148
NODE_16.g4509	572	99.48	*Aspergillus uvarum* CBS 121591 XP_025491187
NODE_16.g4520	585	92.31	*Aspergillus brunneoviolaceus* CBS 621.78 XP_025439954
NODE_18.g4952	739	99.30	*Aspergillus uvarum* CBS121591 XP_025493482
NODE_55.g9244	578	97.75	*Aspergillus uvarum* CBS 121591 XP_025487768.1
NODE_57.g9409	545	100	*Aspergillus uvarum* CBS 121591 XP_025487823
NODE_60.g9637	584	100	*Aspergillus uvarum* CBS 121591 XP_025496649.1
NODE_70.g10053	522	100	*Aspergillus uvarum* CBS 121591 XP_025489644.1
NODE_71.g10109	532	96.75	*Aspergillus uvarum* CBS 121591 XP_025495855.1
NODE_98.g10681	505	88.20	*Penicillium angulare* N7456_012083 KAJ5088467

**Table 2:** Biosynthetic gene clusters (BGCs) identified by antiSMASH with 100% similarity to known metabolite pathways.

Region	Type	Metabolite	Reported Bioactivity	Potential Application
3.1	Terpene	Clavaric acid	Farnesyltransferase inhibition [29]	Anticancer therapeutics
3.2	T1PKS	Naphtho-γ-pyrone	Antimicrobial [30]	Food preservatives, antibiotics
6.1	NRPS	Cyclic depsipeptides	Insecticidal, antimicrobial, anticancer [31]	Biopesticides, drug discovery
10.1	Indole	Okaramine D	Insecticidal [32]	Agricultural biocontrol
21.4	Indole, NRPS, NRPS-like (híbrido)	histidyltryptophanyldiketopiperazine/roquefortine C/roquefortine D/meleagrine/glandicoline A/B/	Neurotoxicity (roquefortines), antimicrobial, cytotoxic, acetylcholinesterase inhibition (meleagrine, glandicolines) [33]	Natural product scaffolds for drug discovery; neuropharmacology; antimicrobial agents (limited due to toxicity)
59.1	T1PKS	Monascorubrin	Pigmentation, antioxidant [34]	Natural food colorants

**Table 3:** Proteins identified by nano LC–MS/MS analysis of the tannase-active band from *Aspergillus uvarum* HT4.

Identifier	Unique Peptides	Spectrum Count	% Coverage	Protein Score	Function
NODE_18.g4952	17	93	21.4	46.99	tannase
NODE_60.g9637	13	41	15.4	35.50	tannase
NODE_43.g8261	11	30	16.2	25.57	alpha/beta-hydrolase
NODE_58.g9479	8	14	24.2	23.12	alpha-L-arabinofuranosidase
NODE_44.g8317	1	12	3.9	3.94	translation elongation factor EF-Tu
NODE_25.g6245	4	11	6.6	11.21	glucanase
NODE_23.g5957	4	9	6.5	6.95	uncharacterized protein
NODE_86.g10522	2	3	5.7	4.28	alpha/beta-hydrolase

## Data Availability

Genome sequence of *A. uvarum* HT4 is available at DDBJ/ENA/GenBank under the accession JBLHEK000000000. The mass spectrometry proteomics data have been deposited to the ProteomeXchange Consortium via the PRIDE 1 partner repository with the dataset identifier PXD066288. The raw data supporting the conclusions of this article will be made available by the authors on request.

## Supplementary Materials

The following supporting information can be downloaded at: https://www.mdpi.com/article/10.3390/jof11100722/s1, Table S1. Sequences of extracellular tannases expressed by *A. uvarum.* Table S2. Secretome of HT4: extracellular proteins and their relative abundance (NSAF). Table S3. List of all 60 predicted biosynthetic gene clusters, including cluster types, genomic positions, and product prediction.

## Abbreviations

The following abbreviations are used in this manuscript:

TE	Tannase extract
BGCs	Biosynthetic gene clusters
