# Supplementary material for: From Isolation to Genomics: Characterization of Aspergillus uvarum HT4 as a Novel Producer of Extracellular Tannase
Source: J Fungi (Basel). 2025 Oct 7;11(10):722. doi: 10.3390/jof11100722 (PMC12565382; doi:10.3390/jof11100722)
Supplement: Supplementary file 1 [file jof-11-00722-s001.zip › jof-3838248-supplementary.pdf]

Table S1 Sequences of extracellular tannases expressed by *A. uvarum.*

Expressed extracellular tannases	Sequences
**NODE 18 length 653725 cov 92.2.g4952**	MRSFSWVPLTGLAALAHGASLSDICTTSYATTAFPATDLIPGITLDPSSITTALVTNSS<br>VTSDFYPSSVIDYCNVTFAYSHNGRDDQVYLQIWLPDPSIFQNRWLSTGGGGYAIN<br>SGSSSLPGGIIYGAASGLTDGGFGSFDTNADAAMLLANYTLDYETLYMFAYKAHW<br>ELTKIGKAFTRNVYNVTDDEKLYAYYQGCSEGGREGWSQVQRYGDEWDGAAIGA<br>PAFRWSFQQTQHLWSNVVEQTLDYYPPPCELEKIVNATIAACDPLDGKTDGVVAR<br>SDLCALHFNISSLIGEAYSCAATAGSRFSSATPAQNGTVSAQGVQVAQTILDGAHD<br>SAGKRVYFSYQPAAAFDDAATQYNSTTGEWGLEIDQLGGEHIGLLVYENGTTLDS<br>LAGVTYDTLKDWMISGLLQYYSTLQTTWPDLTPFHAAGGKVIHFHGEADASIPTA<br>SSVRYWESVRSVMYPSLSYNASADALNDWYRLFLVPGAAHCATNDAMPNGPFP<br>NKNLEVLIDWVENGIVPVTLNGTVLQGDNEGQEQQICAWPLRPLWNGDDLECV<br>YDQASIDTWHFDLNAVPIPVY
**NODE 60 length 181120 cov 91.6.g9637**	MGKYSRAAVAALAAATANATSLSDLCTVSNVQSALPSNGTLLGINMIPSSVTAGV<br>TTSSGGMGGGMKRADSYTYCNVTVAYTHTGKGDKVVLKYAFPSPSEFKNRFYVA<br>GGGGFSLSSDATGGLEYGAASGATDAGYDAFSYSYDEVVLYGNGSINWDATYMF<br>GYQALGEMTQIAKPLARGFYGLDSGKKIYTYYEGCSDGGREGMSQVQRWGSEYD<br>GAITGAPAFRFAQQQVHHVFSSAVEHTMDYYPEPCMLDKIVNATIEACDPLDGRT<br>DGVISRTDLCMLNFNLTSVIGESYYCAEQNYTSLGFGFSKRAEGSSTSYQPAQNGT<br>VTAEGVKLAQAIYDGLHNSQGERAYLSWQIGSELSDATTEYNSDTGAWELDIPST<br>GGEYVTKFVQLLNIDNLSNLDNVTYDTLVGWMNTGMIRYIDSLQTTIPDLTEFESS<br>GGKLLHYHGESDPSIPAASSVHYWQAVRSIMYGDLSYQESLQKMADWYQFYLVP<br>GAAHCGTNSLQPGPYPEDNMEIMINWVENGVKPSRLNATVSSGSYAGETQMLC<br>QWPTRPLWSSNSSFSCVHDQKSIDSWTYEFPAFKLPVY

Table S2. Secretome of HT4: extracellular proteins and their relative abundance (NSAF)

Extracellular proteins of HT4	Average of NSAF
Uncharacterized protein	0.004108225
Protein EPD1 precursor	0.007612233
Concanavalin A-like lectin/glucanase	0.002090933
Tannase and feruloyl esterase	0.021698425
Acid protease	0.003459325
Xyloglucan-specific endo-beta-1,4-glucanase precursor	0.00831025
alpha-L-arabinofuranosidase	0.003811125
Putative tannase	0.0138239
Uncharacterized protein	0.003195475
Putative carboxylesterase	0.001360933
Uncharacterized protein	0.0022296
Cysteine hydrolase family protein	0.0024452
Endo-polygalacturonase A pgaA	0.008419775
Serine carboxypeptidase pepF	0.000839325
GPI anchored protein	0.00140075
Ribonuclease M	0.00669295

Table S3. List of all 60 predicted biosynthetic gene clusters, including cluster types, genomic positions, and product prediction.

Region	Type	Genomic Location Start – End (nt)	Most similar known cluster	Similarity
1.1	NRPS-like	1,160,079 - 1,199,747	-	-
1.2	NRPS-like	1,402,335 - 1,441,679	-	-
1.3	T1PKS	1,554,872 - 1,600,008	-	-
1.4	T1PKS, NRPS (híbrido)	1,852,339 - 1,964,021	Asperphenamate	75%
2.1	NRPS-like	214,456 - 248,920	-	-
2.2	NRPS	870,590 - 933,218	-	-
3.1	Terpene	115,189 - 139,781	clavaric acid	100%
3.2	T1PKS	270,694 - 317,010	Naphthopyrone	100%
5.1	NRPS-like	97,493 - 141,793	-	-
6.1	NRPS	753,858 - 812,553	cyclo-(D-Phe-L-Phe-D-Val-L-Val) / cyclo-(D-Tyr-L-Phe-D-Val-L-Val) / etc.	100%

Region	Type	Genomic Location Start – End (nt)	Most similar known cluster	Similarity
9.1	T1PKS	48,310 - 120,582	nivalenol / deoxynivalenol	8%
10.1	Indole	86,751 - 108,629	okaramine D	100%
10.2	T1PKS	401,273 - 449,030	Epipyriculol	17%
11.1	NRPS-like	155,353 - 189,653	-	-
11.2	Indole	436,090 - 457,578	-	-
11.3	NRPS	585,040 - 628,635	-	-
12.1	T1PKS	207,786 - 256,244	-	-
16.1	NRPS, T1PKS (híbrido)	184,417 - 262,915	NG-391	33%
16.2	NRPS-like	269,190 - 310,165	-	-
16.3	Terpene	478,156 - 499,999	-	-

Region	Type	Genomic Location Start – End (nt)	Most similar known cluster	Similarity
16.4	NRPS-like	627,207 - 671,324	-	-
17.1	NRPS, T1PKS (híbrido)	465,189 - 576,654	echinocandin B	61%
18.1	T1PKS	200,194 - 245,633	Patulin	66%
19.1	NRPS	139,798 - 180,280	-	-
19.2	NRPS	312,761 - 379,049	Aspercryptins	53%
19.3	NRPS	389,978 - 433,329	-	-
19.4	NRPS	596,101 - 642,595	griseofulvin / epidechlorogriseofulvin / etc.	9%
21.1	Siderophore	47,140 - 60,003	-	-
21.2	NRPS-like	93,817 - 129,507	-	-
21.3	T1PKS, Terpene (híbrido)	147,310 - 206,261	-	-

Region	Type	Genomic Location Start – End (nt)	Most similar known cluster	Similarity
21.4	Indole, NRPS, NRPS-like (híbrido)	208,325 - 280,022	histidyltryptophanyldiketopiperazine / roquefortine D / etc.	100%
24.1	NRPS-like	1 - 24,115	-	-
24.2	T3PKS	37,433 - 78,150	-	-
24.3	Terpene	357,291 - 381,568	-	-
25.1	NRPS, NRPS-like	321,717 - 391,025	-	-
28.1	Terpene	185,714 - 208,242	-	-
34.1	Betalactone	295,253 - 324,510	-	-
35.1	NRPS-like, NRPS	131,999 - 216,527	-	-
38.1	NRPS-like, NRPS	165,234 - 210,683	-	-
39.1	T1PKS	311,487 - 359,278	-	-

Region	Type	Genomic Location Start – End (nt)	Most similar known cluster	Similarity
44.1	NRPS	176,312 - 229,895	-	-
45.1	Terpene	133,559 - 154,736	-	-
48.1	Indole	108,783 - 130,123	-	-
49.1	NRPS-like	1 - 40,536	-	-
50.1	T1PKS	240,298 - 272,450	4-epi-15-epi-brefeldin A	20%
52.1	T1PKS, NRPS (híbrido)	64,201 - 150,189	Neosartorin	47%
52.2	T1PKS	187,898 - 236,460	-	-
54.1	NRPS	9,036 - 63,627	-	-
55.1	NRPS-like	197,793 - 236,567	-	-
56.1	NRPS-like	159,789 - 204,202	-	-
57.1	NRPS	177,666 - 233,626	-	-

Region	Type	Genomic Location Start – End (nt)	Most similar known cluster	Similarity
58.1	T1PKS	85,629 - 137,268	-	-
58.2	Terpene	152,080 - 173,688	squalestatin S1	40%
59.1	T1PKS	33,107 - 89,440	Monascorubrin	100%
61.1	T1PKS	121,380 - 168,690	Trypacidin	57%
62.1	NRPS-like	43,315 - 86,338	-	-
63.1	NRPS, T1PKS (híbrido)	3,775 - 101,108	Chaetoglobosins	57%
86.1	NRPS	26,895 - 72,163	-	-
91.1	NRPS	40,994 - 72,095	cyclopiazonic acid	57%
101.1	NRPS-like	5,153 - 32,608	-	-
